# SelO-mediated NAD^+^ hydrolysis safeguards mitochondrial homeostasis

**DOI:** 10.1186/s43556-026-00509-1

**Published:** 2026-07-22

**Authors:** Tong Yao, Shuai Wang, Fangfang Zhou

**Affiliations:** Children’s Hospital of Soochow University, Jiangsu Key Laboratory of Infection and Immunity, Institutes of Biology and Medical Sciences, Suzhou Medical College of Soochow University, Suzhou, 215025 China

A recent paper published in *Cell* by Jia et al. [1] identifies SELENOO (SelO) as a metabolically responsive mitochondrial NADase. Upon elevation of mitochondrial matrix pH, SelO is activated and hydrolyzes Nicotinamide Adenine Dinucleotide (NAD^+^) to Nicotinamide Mononucleotide (NMN) and Adenosine monophosphate (AMP), thereby decreasing local NAD^+^ availability and fatty acid β-oxidation (FAO). Through this mechanism, SelO protects mitochondrial homeostasis from sustained metabolic stress.

Previous studies have demonstrated, using in vitro radiolabeling experiments, that SelO mediates AMPylation of redox homeostasis-related proteins, transferring AMP from ATP to serine, threonine, and tyrosine residues on its substrates [2]. Novelly, Jia et al. uncovered that SelO also possesses intrinsic NADase activity, revealing an additional function for this pseudokinase. NAD^+^ is a central metabolic cofactor and is indispensable for mitochondrial respiration and redox homeostasis. Although the pathways responsible for NAD^+^ replenishment have been largely defined—including Solute Carrier Family 25 Member 51 (SLC25A51), which serves as the principal transporter for direct import of cytosolic NAD^+^ into the mitochondrial matrix [3], and Nicotinamide nucleotide adenylyltransferase 3 (NMNAT3)-mediated intramitochondrial NAD^+^ synthesis—the mechanisms underlying active NAD^+^ degradation within mitochondria remain poorly understood. Given that mitochondrial NAD^+^ levels can undergo rapid and pronounced fluctuations under metabolic stress, and the known broad-spectrum NAD^+^-consuming enzymes (e.g., SARM1, PARPs, and sirtuins) cannot fully account for these dynamic changes, the identification of a dedicated mitochondrial NADase has emerged as a critical and outstanding question in the field.

The study begins with an in silico reverse-docking screen designed to identify mitochondrial NAD-binding proteins. Among the candidates, SelO stood out because its depletion elevated the levels of both total cellular NAD^+^ and mitochondrial NAD^+^, suggesting that SelO normally promotes NAD^+^ consumption. The authors subsequently purified recombinant SelO and showed biochemically that SelO directly hydrolyzes NAD^+^ to generate NMN and AMP. Importantly, this reaction was Mn^2+^-dependent, obeyed Michaelis–Menten kinetics, and did not proceed efficiently with other structurally related cofactors, establishing that SelO acts as a selective NADase rather than a nonspecific hydrolase [1].

To determine how SelO catalyzes this reaction, the authors crystallized and solved the structure of ydiU, a deeply conserved *E. coli* homolog of SelO. By combining structural comparison, AlphaFold-based modeling, and mutational analyses, they found that the conserved C-terminal selenocysteine-serine-serine (CSS) tail of SelO is essential for NAD^+^ hydrolysis. In particular, the C667 of SelO appears to be a critical catalytic residue positioned adjacent to the scissile bond of NAD^+^ but dispensable for its AMPylation activity. The C667A mutation markedly impaired SelO NADase activity, suggesting that the C-terminal tail of SelO is required for its catalytic activation. Moreover, SelO consistently shows higher enzymatic activity at alkaline matrix pH, a condition that can arise from multiple factors. Inhibition of proton re-entry via H⁺-ATP synthase is a notable cause of this pH shift, and such alkalization serves as a hallmark of mitochondrial stress [4]. This suggests that SelO-mediated NAD⁺ hydrolysis represents a mechanism for preserving mitochondrial homeostasis (Fig. 1) [1].

**Fig. 1:**
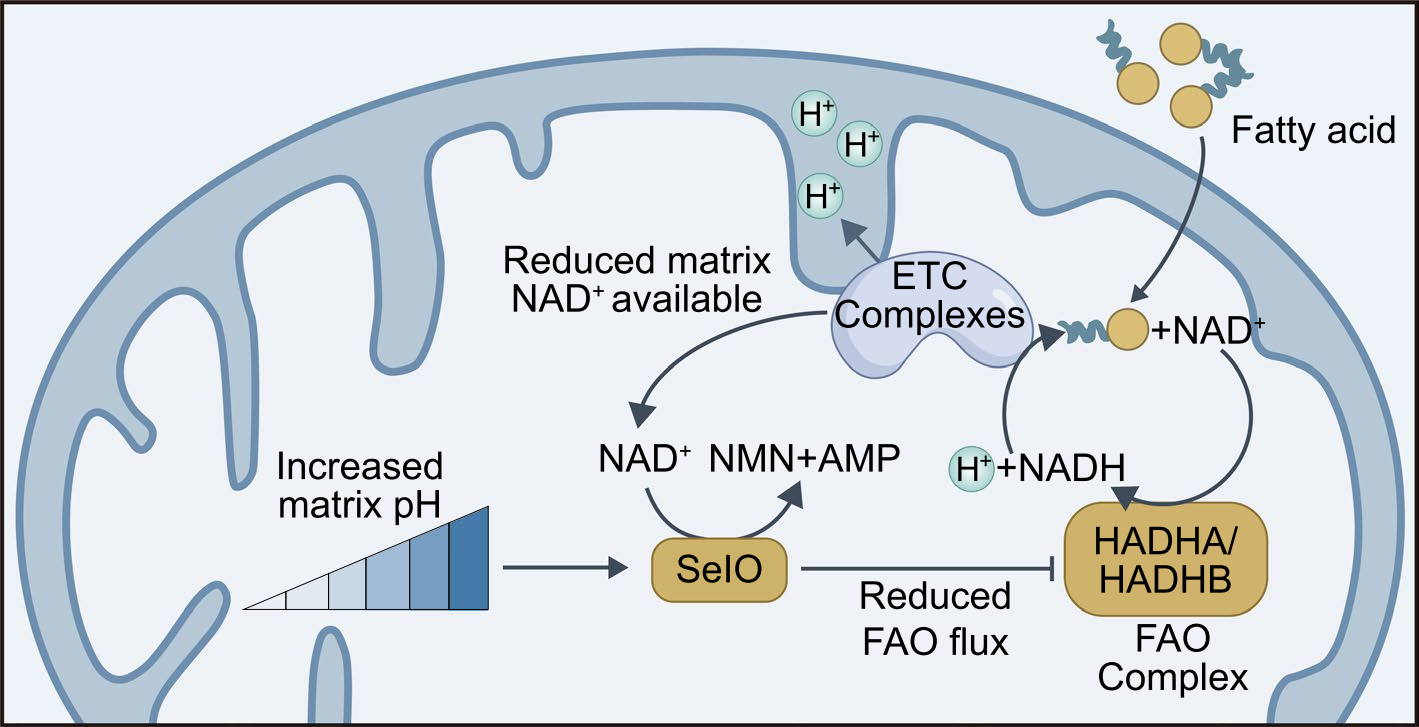
SelO-mediated NAD^+^ hydrolysis suppresses fatty acid β-oxidation and maintains mitochondrial homeostasis. Enhanced mitochondrial respiration drives H⁺ efflux from the matrix into the intermembrane space via ETC complexes, causing matrix alkalinization and subsequent SelO activation. SelO hydrolyzes NAD⁺ to NMN and AMP, thereby reducing local NAD⁺ availability and FAO flux. This metabolic constraint alleviates excessive mitochondrial stress and contributes to the preservation of mitochondrial homeostasis

Given that mitochondrial NAD^+^ metabolism is intimately coupled with energy utilization, the authors next evaluated the metabolic consequences of SelO deficiency in vivo. They found that liver-specific deletion of SelO elevated hepatic mitochondrial NAD^+^ levels and diminished NMN level. Transcriptomic and metabolomic analyses further revealed that FAO pathway was markedly upregulated in the absence of SelO. Moreover, under high-fat diet conditions, SelO-deficient mice exhibited attenuated hepatic lipid accumulation and enhanced lipid utilization. Collectively, these results indicate that SelO-mediated NAD^+^ hydrolysis normally functions to constrain lipid catabolism [1]. Nevertheless, the observation of increased FAO intermediates raises the possibility of alternative explanations, such as bottlenecks within the FAO pathway and rather than enhanced FAO flux. This constitutes an interesting question for future investigation.

To understand how SelO regulates fatty acid oxidation, the authors performed protein interaction and enzymatic activity assays. SelO was revealed to directly associate with and suppress key FAO enzymes through NAD^+^ depletion, specifically mitochondrial trifunctional protein (MTP) complex HADHA and HADHB, which catalyze crucial steps in β-oxidation. Consistently, the *E. coli* homolog ydiU also binds to the bacterial trifunctional enzyme complex fadA (Fatty acid oxidation complex subunit alpha)/fadB. However, the precise impact of SelO on the HADHA/HADHB complex remains to be elucidated. NAD^+^ is required for HADHA catalysis, and its depletion inhibits the HADHA activity. Addition of SelO, but not the C667A mutant, inhibits MTP complex activity in vitro, indicating that SelO limits FAO flux through local NAD^+^ depletion [1]. Nevertheless, the FAO fluxes have not been directly measured in vivo. Future studies may benefit from directly assessing FAO flux via isotope-labeled fatty acids and distinguishing total NAD⁺ depletion from NAD⁺/NADH ratio change.

Finally, the authors examined the physiological importance of SelO in maintaining mitochondrial homeostasis. Knockout of SelO reduces hepatic lipid accumulation in mice, and causes mitochondrial dysfunction, including mitochondrial fragmentation, condensed matrix, expanded intracristae space, outer mitochondrial membrane defect, and TOM20-HSP60 interaction. These mitochondrial dysfunctions were rescued by reintroduction of WT SelO but not by the NADase catalytic mutant, confirming the protective effect of SelO NADase activity on mitochondrial homeostasis. Together, these data indicate that SelO does not simply suppress metabolism but actively safeguards mitochondria from damage caused by excessive metabolic activation (Fig. 1) [1]. Nevertheless, the direct causal link between a reduced NAD⁺ pool and mitochondrial homeostasis remains to be elucidated.

This study identifies SelO as a crucial NAD^+^-degrading enzyme that plays a critical regulatory role in FAO and mitochondrial homeostasis. Although the underlying mechanism is still unclear, emerging evidence demonstrates that aging is closely associated with suppressed FAO and reduced NAD^+^ levels, and these metabolic changes are key drivers of age-related diseases. Mitochondrial FAO dysregulation is increasingly recognized as a hallmark of aging-related pathologies and a defining feature of senescent cells. In senescent cells and the liver of aged animal, FAO associated proteins are significantly downregulated, leading to the accumulation of fatty acyl-CoAs and acylcarnitines. These metabolites drive lipotoxicity, mitochondrial dysfunction, and metabolic rigidity, which are key contributors to tissue aging and organ failure. Furthermore, impaired FAO has been shown to promote cellular senescence in both cellular and animal models by facilitating lipid accumulation and the senescence-associated secretory phenotype (SASP). Conversely, restoring FAO prevents senescence and preserves mitochondrial function [5]. In this paper, fragmented mitochondria, condensed mitochondrial matrix, and expanded intracristae space were observed in the livers of SelO knockout mice. These morphological abnormalities are associated with SelO's role in lipid oxidation and are exacerbated in aged mice, suggesting a potential role for SelO in cellular senescence and aging. Additionally, the long-term effects of NAD^+^ depletion, particularly on aging, senescence and metabolic diseases remain to be defined.

This article raises several unresolved questions. Although SelO reduces the matrix NAD⁺ pool, how this decrease alleviates mitochondrial stress remains unclear. Under physiological conditions, enhanced ETC drives coupled proton re-entry via ATP synthase, tightly regulating matrix pH; elevated pH typically indicates ATP synthase dysfunction and ETC inhibition. Thus, the physiological significance of SelO-mediated additional "braking" under these conditions remains to be clarified. Futhermore, other NAD^+^-dependent enzymes such as sirtuins and PARPs have long been implicated in metabolic stress responses, but their interplay with SelO-mediated NAD^+^ degradation remains unexplored. How these distinct NAD^+^-dependent pathways converge to regulate mitochondrial function is an exciting direction for future research.

In conclusion, Jia et al. uncover a new regulatory axis in mitochondrial NAD^+^ metabolism that serves as a safeguard mechanism against mitochondrial stress. This study identifies SelO as a previously unrecognized mitochondrial NAD^+^-degrading enzyme. SelO-mediated NAD^+^ hydrolysis is revealed to suppress FAO by locally controlling NAD^+^ availability. Finally, SelO is demonstrated to be essential for protecting mitochondrial homeostasis against sustained metabolic overactivation. These findings raise intriguing questions about the broader implications of mitochondrial NAD^+^ degradation in aging, senescence, and metabolic diseases, while also suggesting potential therapeutic targets.

## Data Availability

Not applicable.
